# Evaluation of Missed Radiological Diagnosis in Multiple Trauma Patients With Full-Body Computed Tomography in the Emergency Department

**DOI:** 10.7759/cureus.51621

**Published:** 2024-01-03

**Authors:** Hakan Selçuk, Nese Oray, Recep M Mert, Hüseyin Odaman, Handan Güleryüz

**Affiliations:** 1 Emergency Department, Babaeski State Hospital, Kırklareli, TUR; 2 Emergency Medicine, Dokuz Eylül University Faculty of Medicine, Izmir, TUR; 3 Emergency Medicine, Dokuz Eylül University Hospital, Izmir, TUR; 4 Radiology, Dokuz Eylül University Hospital, Izmir, TUR

**Keywords:** misdiagnosis, radiological diagnoses, computed tomography, full-body computed tomography, multiple trauma, emergency department

## Abstract

Introduction: This observational, cross-sectional, and retrospective study was conducted at the Dokuz Eylül University Emergency Department in İzmir, Turkey, after obtaining ethical consent (Dokuz Eylül University Medical Faculty Ethics Committee, approval no. 2019/15-37). In this study, we aimed to determine missed radiological diagnoses and their effects on mortality and morbidity by comparing the ED diagnoses of patients and radiology reports of these patients who presented to the emergency department (ED) with multiple traumas and scanned full-body computed tomography (CT).

Materials and methods: This observational, cross-sectional, and retrospective study was conducted at the Dokuz Eylül University Emergency Department in İzmir, Turkey. Adult patients who presented to the ED with trauma between July 1, 2016 and June 30, 2018 and who had a full-body CT were included in the study. Radiology reports of CTs and ED electronic file information were compared. Missed diagnoses were determined for all body parts.

Results: In this study, 1,358 patients who had scanned full-body CT in the ED were evaluated. A total of 369 diagnoses were missed in 248 (18.3%) of the patients. The diagnosis-to-patient ratio was 0.27. In the process of individually evaluating pathological diagnoses in all body regions, it was low only in brain edema, pneumomediastinum, bladder injury, and mesentery injury. At least, there was one missed diagnosis in 88 (9.7%) of 907 (66.8%) discharged patients. At least, there was one missed diagnosis in 18/23 (78.3%) patients who died within the first 48 hours. Among the patients who have missed diagnosis, the rate of the discharged patients was 35.5%, patients called back from home was 1.2%, intensive care unit admission was 20.2%, hospitalization was 65.7%, and death was 8.9%. Among the patients who did not have missed diagnosis, the rates were 73.8%, 0%, 5%, 26.9%, and 0.8%, respectively.

Conclusion: Thoracic region pathologies are the most frequently missed pathologies, and orthopedics was the most frequently consulted department related to the missed diagnoses. Patients who have a missed diagnosis had lesser discharging from the ED than the other patients and had higher rates of in-hospital deaths, hospitalization, and intensive care unit admission.

## Introduction

Missing diagnosis or misdiagnosing in multiple trauma patients delays effective treatment, prolongs hospital stay, and increases mortality [1]. In a study examining the misdiagnoses in multiple trauma patients in the literature, the misdiagnosis rate was 16.2%, and the anatomical regions misdiagnosed were the extremities and pelvis, abdomen and pelvic organs, and thoracic regions, in order of frequency [2]. However, with the progression in imaging methods, it has been reported that the rate of misdiagnosis of vascular and heart injuries has increased [3,4,5].

Brain, cervical, thorax, and abdomen imaging is often performed together in patients who present with high-energy multiple trauma to the emergency department (ED), and computed tomography (CT) is preferred as the imaging method. In this study, we compared emergency department diagnoses (EDDs) and diagnoses in radiology report (RDs) in multiple trauma patients with full-body CT. We aimed to identify missed radiological diagnoses and determine the effects of these diagnoses on mortality and morbidity.

## Materials and methods

This observational, cross-sectional, and retrospective study was conducted at the Dokuz Eylül University Emergency Department in in İzmir, Turkey, after obtaining ethical consent (Dokuz Eylül University Medical Faculty Ethics Committee, approval no. 2019/15-37). Dokuz Eylül University Hospital Emergency Department is a tertiary ED with 50 beds and has 140,000 patient admissions per year. Patients who were over 18 years old and admitted to the ED due to trauma and had brain, cervical, thorax, and abdomen CT (full-body CT) between July 1, 2016 and June 30, 2018 were included in the study. Patients with incomplete information were excluded from the study.

CTs of the patients were taken with the Aquilion Prime device (Toshiba, Japan). Section thickness was provided as 3 mm for brain CT and 2 mm for CT of other areas. A mixture of approximately 70 cc iopromide (1 cc = 370 mg iodine) and 30 cc 0.9% saline was used in patients using contrast media. In contrast-enhanced imaging, first, brain and cervical CTs were taken, and then contrast-enhanced thorax and abdomen CTs were taken.

Official RDs and ED physician’s observation notes and consultation notes recorded from the hospital information database system were assessed by the study team. Age, gender, mechanism of trauma, EDDs, and RDs of the patients were recorded. For mortality, death was evaluated within 48 hours, and for morbidity, hospitalization, admission to intensive care, and the need for medical/surgical additional intervention after evaluation in the ED were evaluated. Considering the possibility of an error in the ED records or RDs, the records with disconcordance between EDDs and RDs were re-evaluated by the study team consisting of emergency medicine and radiology physicians. If there was a material error in the reports, these errors were corrected.

Our primary purpose in conducting this study is to detect missed radiological diagnoses in the ED in multiple trauma patients with full-body CT, and our secondary goal is to determine the effect of missed radiological diagnoses on mortality and morbidity in multiple trauma patients.

The data were evaluated in IBM SPSS Statistics for Windows, version 24 (released 2016; IBM Corp., Armonk, New York, United States) package program. In the descriptive information, those that the data normally distributed were defined as mean ± standard deviation and minimum-maximum value, and those that did not normally distribute were indicated by median and interquartile range (IQR). The variables defined by counting were compared with the chi-square and Fisher's exact test. The variables defined by measurement were compared with the t-test or Mann-Whitney U test according to their adequacy for normal distribution. While evaluating whether there is a difference between RDs and EDDs with the McNemar test, overlaps between radiological diagnosis and emergency room diagnosis were measured by kappa analysis. In the Kappa analysis, 0-0.20 points were evaluated as a slight agreement, 0.21-0.40 points as fair, 0.41-0.60 points as moderate, 0.61-0.80 points as substantial, and 0.81-1.00 points as an almost perfect agreement. The significance level was accepted as p < 0.05.

## Results

The mean age of the 1,358 patients included in the study was 45.9 ± 1.1 years (range: 18-99). The most common trauma mechanisms were traffic accident (n = 433, 31.9%), motorcycle accident (n = 213, 15.7%), falling at the same level (n = 194, 14.3%), non-pedestrian traffic accident (n = 193, 14.2%), fall from height (n = 177, 13.0%), and other reasons (n = 148, 10.9%).

Brain CT imaging outcomes

The presence of any pathology in 166/191 patients (86.9%), any intracranial bleeding in 138/152 patients (90.8%), and any cranial fracture in 83/98 patients (84.7%), cerebral edema in 9/38 patients (23.6%), cerebral shift in 15/19 patients (78.9%), intracranial foreign body in 3/3 patients (100%), pneumocephalus in 30/33 patients (90.9%), and subcutaneous foreign body diagnoses in 8/20 patients (40%) were correctly detected in the EDDs in accordance with the RDs.

There was an "almost perfect agreement" between the RDs and EDDs in detecting the presence of any pathology in brain CT imaging, any intracranial hemorrhage (epidural hematoma, subdural hematoma, subarachnoid hemorrhage, intraventricular hemorrhage, and contusion diagnosis), and any fracture (any of the diagnoses of partial/displaced fracture and linear fracture presence). Weak overlapping was found in the diagnosis of cerebral edema. Except for contusion, cerebral edema, and subcutaneous foreign body diagnoses, there was no difference between the RDs and EDDs on brain CT imaging results (Table 1, p* values).

**Table 1:** Brain CT imaging outcomes * McNemar test ⱡ󠆁 kappa analysis; EDD: emergency department diagnosis; RD: radiology report

Diagnosis		RD +	RD -	p*	Kappa	pⱡ󠆁
Presence of any pathology	EDD +	166	26	1.000	0.845	<0,001
	EDD -	25	1141			
Intracranial hemorrhage	EDD +	138	22	0.243	0.870	
	EDD -	14	1184			
Contusion	EDD +	31	11	0.014	0.606	
	EDD -	27	1289			
Epidural hematoma	EDD +	21	9	0.607	0.731	
	EDD -	6	1331			
Subdural hematoma	EDD +	56	11	0.200	0.777	
	EDD -	19	1272			
Intracerebral hematoma	EDD +	14	8	0.791	0.661	
	EDD -	6	1330			
Subarachnoid hemorrhage	EDD +	89	19	1.000	0.809	
	EDD -	19	1231			
Intraventricular hemorrhage	EDD +	9	3	0.508	0.663	
	EDD -	6	1340			
Presence of fracture	EDD +	83	12	0.701	0.850	
	EDD -	15	1248			
Linear fracture	EDD +	65	16	1.000	0.785	
	EDD -	17	1276			
Comminuted/displaced fracture	EDD +	38	2	1.000	0.936	
	EDD -	3	1315			
Shifting	EDD +	15	2	0.688	0.831	
	EDD -	4	1337			
Pneumocephaly	EDD +	30	5	0.727	0.879	
	EDD -	3	1320			
Cerebral edema	EDD +	9	1	0.000	0.368	
	EDD -	29	1319			
Subcutaneous foreign body	EDD +	8	0	0.000	0.568	
	EDD -	12	1338			
Intracranial foreign body	EDD +	3	0	1.000	1.000	
	EDD -	0	1355			
Herniation	EDD +	0	1	0.625	-0.001	0.962
	EDD -	3	1354			

**Figure 1:**
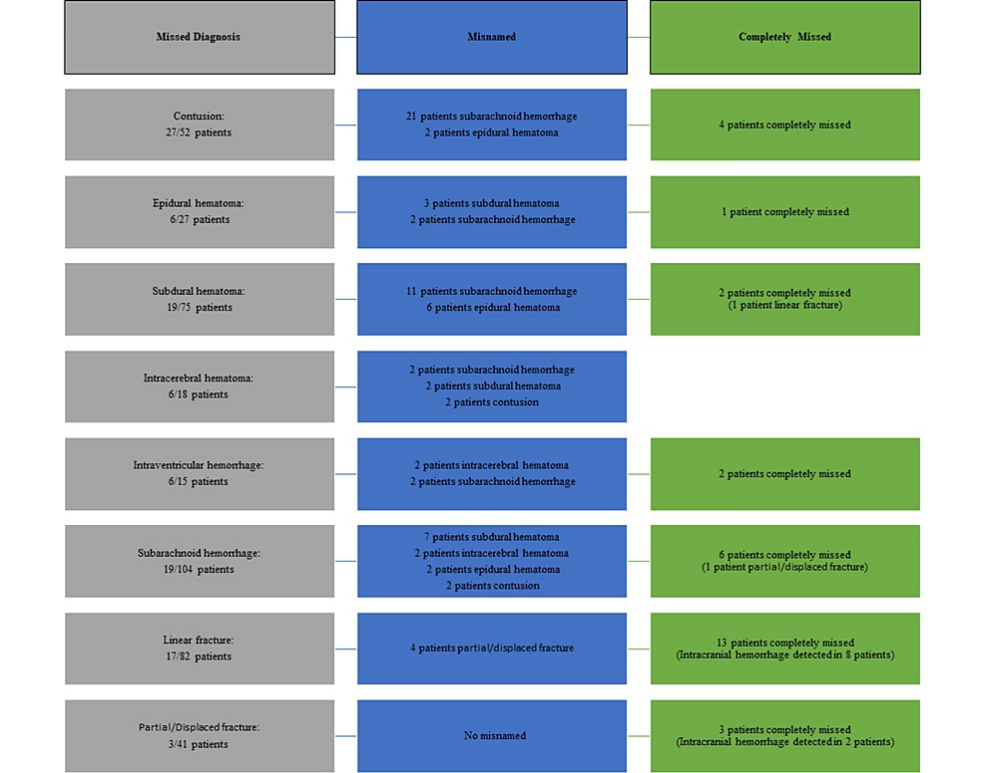
Assessment of discordant diagnoses in brain CT imaging.

Diagnoses that were reported to have RDs but were not detected in the EDD were examined; it was noticed that many diagnoses were misnamed (Figure 1).

Cervical CT imaging outcomes

The presence of any pathology in 44/56 patients (78.6%), C1 fracture in 6/8 patients (75%), C2 fracture in 6/8 patients (75%), C3-7 vertebral fracture 30/39 patients (76.9%), occipital condyle fracture in 3/6 patients (50%), facet dislocation in 4/4 patients (100%), and cervical vertebra dislocation in 2/2 patients (100%) were detected correctly in the ED. Although it was not included in the RDs, it was thought that 18 patients had cervical vertebra fractures, and one patient had an atlantooccipital dislocation and anterior teardrop injury in the ED.

A substantial and almost-perfect agreement was found between the EDDs and RDs of any pathology, C1 fracture, C2 fracture, C3-7 fracture, facet dislocation, occipital condyle fracture, and foreign body in the cervical region (Table 2). There was no difference between the EDDs and RDs in all diagnoses on cervical CT imaging except cervical spinous process fracture (Table 2, p* values).

**Table 2:** Servical CT imaging outcomes * McNemar test ⱡ󠆁 kappa analysis; EDD: emergency department diagnosis; RD: radiology report

Diagnosis		RD +	RD -	p*	Kappa	pⱡ󠆁
Presence of any pathology	EDD +	44	20	0.215	0.721	<0.001
	EDD -	12	1282			
C1 fracture	EDD +	6	2	1.000	0.749	
	EDD -	2	1348			
C2 fracture	EDD +	6	3	1.000	0.704	
	EDD -	2	1347			
C 3-7 fracture	EDD +	30	16	0.230	0.696	
	EDD -	9	1303			
Corpus fractures	EDD +	9	4	1.000	0.689	
	EDD -	4	1341			
Lamina fractures	EDD +	12	1	0.625	0.856	
	EDD -	3	1342			
Spinosus process fractures	EDD +	10	10	0.002	0.663	
	EDD -	0	1338			
Transverse process fractures	EDD +	6	3	1.000	0.629	
	EDD -	4	1345			
Facet dislocation	EDD +	4	0	1.000	1.000	
	EDD -	0	1354			
Occipital condyle fracture	EDD +	3	0	0.250	0.666	
	EDD -	3	1352			

Thorax CT imaging outcomes

In the thorax CT images, 209/265 patients (78.8%) had any lung pathology, 115/145 patients (79.3%) had pneumothorax, 73/103 patients (70.8%) had hemothorax, 105/188 patients (55.9%) had contusion in the lung, 222/262 patients (84.17%) had rib fracture, 72/92 patients (78.3%) had fracture in the thoracic vertebra, 49/55 patients (89.1%) had scapula fracture, 51/57 patients (89.5%) had clavicle fracture, 21/22 patients (95.4%) had humerus fracture, 30/45 patients (66.6%) had sternum fracture, and 3/8 patients (37.5%) had pneumomediastinum. Meanwhile, pericardial effusion was correctly diagnosed in 3/7 patients (42.8%) in accordance with the EDDs and RDs. The EDDs and RDs were consistent in all diagnoses of aortic dissection (n = 3), diaphragm rupture (n = 3), foreign body in thorax (n = 1), thoracic vertebral dislocation (n = 1), foreign body in muscle tissue (n = 1), alveolar hemorrhage (n = 1), and bilateral humeral dislocation (n = 1). Thoracic vertebral lamina fracture in three patients and sternoclavicular dislocationacromioclavicular joint dislocation and bronchial rupture in one patient were missed in the ED.

An almost-perfect agreement (k = 0.829) was found for the presence of any diagnosis between the EDDs and RDs in the thorax CT. The fair agreement was found in the diagnosis of pneumomediastinum (Table 3). There was no difference between the EDDs and RDs in diagnoses other than thoracic pathologies and pneumothorax (Table 3, p* statistics).

**Table 3:** Thorax CT imaging outcomes * McNemar test ⱡ󠆁 kappa analysis; EDD: emergency department diagnosis; RD: radiology report

Diagnosis		RD +	RD -	p*	Kappa	pⱡ󠆁
Presence of any diagnosis	EDD +	383	52	0.842	0,829	<0.001
	EDD -	49	874			
Lung parenchyma pathology	EDD +	209	57	1.000	0.735	
	EDD -	56	1036			
Pneumothorax	EDD +	115	11	0.004	0.832	
	EDD -	30	1202			
Hemothorax	EDD +	73	17	0.079	0.738	
	EDD -	30	1238			
Contusion	EDD +	105	73	0.471	0.507	
	EDD -	83	1097			
Rib fracture	EDD +	222	35	0.644	0.821	
	EDD -	40	1061			
Thoracic vertebrae fracture	EDD +	72	21	1.000	0.762	
	EDD -	20	1245			
Corpus	EDD +	39	17	0.585	0.711	
	EDD -	13	1289			
Transverse process	EDD +	21	5	0.424	0.745	
	EDD -	9	1323			
Spinosus process	EDD +	20	3	1.000	0.848	
	EDD -	4	1331			
Scapula fracture	EDD +	49	7	1.000	0.878	
	EDD -	6	1296			
Clavicle fracture	EDD +	51	5	1.000	0.898	
	EDD -	6	1296			
Humerus fracture	EDD +	21	0	1.000	0.976	
	EDD -	1	1336			
Sternum fracture	EDD +	30	9	0.307	0.705	
	EDD -	15	1304			
Pneumomediastinum	EDD +	3	5	1.000	0.371	
	EDD -	5	1345			
Pericardial effusion	EDD +	3	2	0.688	0.498	
	EDD -	4	1349			
Aortic dissection	EDD +	3	0	1.000	1.000	
	EDD -	0	1355			
Diaphragmatic rupture	EDD +	3	1	1.000	0.857	
	EDD -	0	1354			

Abdomen CT imaging outcomes

Liver injury in 22/32 patients (68.7%), spleen injury in 23/28 patients (82.1%), kidney injury in 12/19 patients (63.1%), adrenal gland injury in 4/9 patients (44.4%), bladder injury in 1/3 patients (33.3%), retroperitoneal injury in 23/36 patients (63.8%), vascular injury in 4/7 patients (57.1%), intraabdominal free gas in 6/7 patients (85.7%), intraabdominal free fluid in 42/59 patients (71.1%), lumbar vertebral fractures in 117/138 patients (84.8%), sacrum/coccyx fracture in 23/34 patients (67.6%), pelvic fracture in 76/81 patients (93.8%), and femur fracture in 31/34 patients (91.1%) were correctly detected with the abdomen CT imaging in the ED. Among the vertebral fractures, lumbar vertebral corpus fracture in 44/51 patients (86.3%), lumbar vertebral lamina fracture in 2/4 patients (50%), lumbar vertebra transverse process fracture in 84/100 patients (84%), lumbar spine spinous process fracture in 7/10 patients (70%) were diagnosed in the ED in accordance with the RDs. Among the pelvic bones, ilium fracture in 20/25 patients (80%), ischial fracture in 4/8 patients (50%), pubis fracture in 58/65 patients (89.2%), acetabular fracture in 29/34 patients (85.3%), and dehiscence of the sacroiliac joint in 3/4 patients (75%) were diagnosed in the ED in accordance with the RD (Table 4). All diagnoses of femur dislocation (n = 5), intestinal perforation (n = 3), foreign body in two patients, renal cyst rupture and pelvic hematoma, traumatic bowel herniation, perianal injury, pancreatic injury, and diasthesis pubis in one patient were all diagnosed correctly in the ED. On the other hand, diagnoses of perirenal hematoma, ovarian cyst rupture, and penile injury were missed in one patient. Correct diagnosis was made in one each of two patients with psoas hematoma and scrotal injury. An almost-perfect agreement (k = 0.845) was found between the EDDs and RDs in evaluating the presence of any pathology. There was no difference between the EDDs and RDs in all abdominal CT diagnoses, except retroperitoneal hematoma and sacrum/coccyx fracture (Table 4, p* statistics).

**Table 4:** Abdomen CT imaging outcomes * McNemar test ⱡ󠆁 kappa analysis; EDD: emergency department diagnosis; RD: radiology report

Diagnosis		RD +	RD -	p*	Kappa	pⱡ󠆁
Presence of any pathology	EDD +	257	37	0,813	0.845	<0.001
	EDD -	34	1030			
Liver injury	EDD +	22	5	0,302	0.740	
	EDD -	10	1321			
Spleen injury	EDD +	23	7	0,774	0.789	
	EDD -	5	1323			
Pankreatic injury	EDD +	1	1	1,000	0.666	
	EDD -	0	1356			
Kidney injury	EDD +	12	7	1.000	0.626	
	EDD -	7	1332			
Adrenal gland injury	EDD +	4	0	0.063	0.614	
	EDD -	5	1354			
Bladder injury	EDD +	1	3	1.000	0.284	
	EDD -	2	1352			
Retroperitoneal hematoma	EDD +	23	1	0.002	0.762	
	EDD -	13	1321			
Mesenteric injury	EDD +	1	0	0.063	0.285	
	EDD -	5	1352			
Vascular injury	EDD +	4	3	1.000	0.569	
	EDD -	3	1348			
Intraabdominal free gas	EDD +	6	1	1.000	0.856	
	EDD -	1	1350			
Intraabdominal free liquid	EDD +	42	10	0.248	0.746	
	EDD -	17	1289			
Lombar vertebrae fracture	EDD +	117	29	0.193	0.810	
	EDD -	19	1193			
Corpus fracture	EDD +	44	10	0.629	0.832	
	EDD -	7	1297			
Lamina fractures	EDD +	2	1	1.000	0.570	
	EDD -	2	1353			
Transverse process fractures	EDD +	84	21	0.511	0.805	
	EDD -	16	1237			
Spinosus process fractures	EDD +	7	4	1.000	0.664	
	EDD -	3	1344			
Sacrum/coccyx fracture	EDD +	23	2	0.022	0.775	
	EDD -	11	1322			
Presence of pelvic fracture	EDD +	76	5	1.000	0.934	
	EDD -	5	1272			
Ilium fracture	EDD +	20	4	1.000	0.813	
	EDD -	5	1329			
Ischial fracture	EDD +	4	3	1.000	0.531	
	EDD -	4	1347			
Pubic fracture	EDD +	58	1	0.070	0.932	
	EDD -	7	1292			
Acetabular fracture	EDD +	29	4	1.000	0.862	
	EDD -	5	1320			
Dehiscence of the sacroiliac joint	EDD +	3	0	1.000	0.857	
	EDD -	1	1354			
Femur fracture	EDD +	31	0	0.250	0.953	
	EDD -	3	1324			

Missed diagnosis

A total of 369/58,394 (0.6%) diagnoses were missed in 248 (18.3%) of the 1,358 patients included in the study. The diagnosis-to-patient ratio was 0.27. There diagnoses were 83 brain CT pathologies in 73 patients, 19 cervical CT pathologies in 16 patients, 156 thoracic CT pathologies in 126 patients, and 111 abdominal CT pathologies in 84 patients. The number of patients who have a missed diagnosis in the ED and the number and rates of missed diagnoses are shown in Table 5.

**Table 5:** Distribution patients of the correct diagnoses and missed diagnoses in the ED by region

	Patients of the correct diagnoses		Patients of the missed diagnoses	
	n	%	n	%
Brain BT	1285	94.6	73	5.4
Cervikal CT	1342	98.8	16	1.2
Thorax CT	1232	90.7	126	9.3
Abdominal CT	1273	93.7	84	6.2
Total	1110	81.7	248	18.3

Patient outcomes

Among the 1,358 patients included in the study, 907 (66.8%) were discharged, and 88 (9.7%) of these patients had at least one missed diagnosis. Three of these patients were recalled from home for re-evaluation, after realizing that the diagnosis was missed. Among the 23 patients who died within the first 48 hours of the application, 18 (78.3%) were patients who had at least one missed diagnosis. Whether death was related to missed diagnoses could not be examined in our study. The discharge rate of the patients who had a missed diagnosis was 35.5%, the rate of being called from home was 1.2%, the rate of admission to the intensive care unit was 20.2%, the rate of hospitalization was 65.7%, the mortality rate was 8.9%, and these values of those without a missed diagnosis were 73.8%, 0%, 5%, 26.9%, and 0.8%, respectively. It could not be reached to the current data of six patients, and they were excluded from the analysis in mortality and morbidity assessments.

Effect of missed diagnoses on morbidity

Among the 248 patients who have missed diagnoses, 13 (3.8%) of them realized that they have a missed diagnosis after hospitalization and additional medical intervention (six patients on anti-edema treatment, two patients on oxygen support, and one patient on low-molecular-weight heparin, shoulder arm sling, sitting ring, Philadelphia-type cervical collar, and thoracic-lumbar-sacral orthosis (TLSO) corset) was performed. Eight of the patients had additional surgical interventions (four patients had tube thoracostomy, two patients had intracranial decompression surgery, one patient had splenectomy, one patient had femur fracture operation).

## Discussion

The number of studies in the literature about the effect of missed diagnoses on mortality and morbidity in multiple trauma patients with full-body CT is limited [1,3,6,7,8].

Full-body CT is frequently performed on patients with multiple trauma, and the most common trauma mechanism in similar studies in the literature is traffic accidents. In our study, more than half of the patients (61.8%) presented with in-vehicle and pedestrian traffic and motorcycle accidents, and similar results were found in the studies in the literature. In the studies of Banaste et al. and Yang et al., the rate of patients presenting with falling, which is the second most common mechanism, was 20.7% and 12.4%, respectively; in our study, the rate of patients who presented with falling from a height or the same level was 27.3% [2,9].

In our study, 369 diagnoses were missed in a total of 248 (18.3%) patients in the CT images of 1,358 patients. The diagnosis-to-patient ratio was 1.49. This ratio was 1.48 in the study of Yang et al. and 1.4 in the study of Buduhan et al. [2,3]. In the literature review of Pfeifer and Pape, the rate of missed diagnosis varied between 1.3% and 39% [6]. This variable ratio in studies in the literature may depend on many factors, such as the functioning of the emergency health system (Anglo-American and Franco-German models), characteristics of the hospital where the patients are evaluated (such as being a trauma center, being a primary/secondary/tertiary hospital, being a university hospital), or the characteristics of physicians evaluating trauma patients (e.g., emergency medicine specialist, general practitioner, other specialists). Our hospital is a fully comprehensive tertiary university hospital. With these features, it is a reference hospital where especially critical patients with poor general conditions from rural districts and cities are referred. This situation is one of the reasons that increase the mortality and morbidity of our patients. In our study, mortality, hospitalization, and intensive care unit admission rates were higher in patients with missed diagnoses. In the study of Yang et al., as the severity of trauma increased, the more rate of missed diagnosis increased [2]. We could not give any results about the relationship between the trauma score and mortality and morbidity as trauma scoring was not used in our study. However, we think that the rate of critical patients may be higher than those in other hospitals because we are a reference hospital.

According to the anatomical regions, it was found that the rate of missed diagnosis was the highest (9.3%) in the thoracic region and the least (1.2%) in the cervical region. This may be related to more frequent injuries in the thoracic region and more injuries to the thoracic region that need attention. Although there were minor injuries that did not require intervention, such as simple rib fracture and lung contusion in the diagnoses that were overlooked, there were also major pathologies, such as hemothorax and pneumothorax. It shows the need for training on thoracic CT in traumatic patients, as we have mentioned above. Moreover, it will be beneficial to reporting CT imaging results early in trauma patients.

In our study, it was found that the pathologies in thoracic CT, abdominal CT, and brain CT were missed at most. In the study of Yang et al., the most frequently missed diagnosis was the extremity and pelvis (40%), followed by the abdomen and pelvic organs (20%) and thoracic region (14%). We did not evaluate the extremities in our study. In the study of Yang et al., after the extremities were excluded, abdominal and pelvic structures (52%) were the most frequently missed diagnosis, followed by the thoracic region (23%) and head and neck (18%), respectively. In the studies of Buduhan et al. and Kalemoğlu et al., it was reported that the most frequently missed diagnosis is in the head and thoracic region, excluding the extremities [3,8]. In the study of Houshian et al., there was a diagnosis mostly overlooked in the thorax and abdomen after the extremities [10]. The head, thorax, and abdominal regions are the most common fatal injuries in multiple traumas. Therefore, it is important to examine the imaging in detail and to get radiological reports urgently. In both our study and the study of Yang et al., the rate of missed diagnosis in the cervical region was lesser than the other regions (5.1% and 7.5%, respectively). It is an expected result that traumatic cervical pathologies are seen less frequently compared to other regions and missed diagnoses to be proportionally less frequently.

According to the results of brain CT imaging, the detection rate of any intracranial hemorrhage was 90.8%, and the compatibility of RDs and EDDs for intracranial hemorrhage was found almost perfect level (k = 0.870). On the other hand, it was noticed that mistakes can be made about the correct definition of bleeding (such as epidural, subdural, intracerebral, contusion, subarachnoid, and intraventricular), but these mistakes do not lead to clinical consequences that will change patient management. It was noticed that often these patients have intracranial hemorrhage, and they have consulted to the neurosurgery after all. No bleeding was considered in only six patients with subarachnoid hemorrhage, four patients with a cerebral contusion, two patients with subdural hematoma and intraventricular hemorrhage, and one patient with epidural hematoma. A similar situation is valid for the definition of fractures in the cranial bones. While the agreement rate between EDD and RD was almost perfect in recognizing partial/displaced fractures, this ratio was relatively low in linear fractures. On the other hand, linear fractures are often diagnoses that do not change the need for surgical or medical treatment. These results suggest that patients in the ED can be diagnosed with high accuracy.

It was found that there was a slight agreement in the diagnosis of cerebral edema and herniation in brain CT. There were three patients with missed herniation and 29 patients with missed cerebral edema diagnosis. Cerebral edema may be omitted because most of the patients with cerebral edema have other diagnoses, and it is considered relatively unimportant and not written in the file notes or focus on other diagnoses. Emergency physicians and related department consultants evaluating patients in the ED should be more careful in terms of missed diagnoses.

It was observed that injuries, such as C1, C2, cervical vertebra corpus, and occipital condyle fractures, that were diagnosed had a substantial agreement with RD in the ED in cervical CT imaging results. This result is gratifying. We think that early and correct diagnosis of cervical trauma may decrease mortality and morbidity.

Thorax CT imaging results showed that lung contusions were missed more often than pneumothorax and hemothorax. We think that this situation is related with the lesser recording file notes of lung contusions due to their less clinical significance. Accurate evaluation of diagnoses that cause serious mortality and morbidity, such as aortic dissection and diaphragm rupture in all patients, supports this view. In general, the rate of recognizing lung pathologies and their agreement level with RD were found to be substantial, which is also valid for bone structures. On the other hand, the agreement level between the diagnosis of pneumomediastinum in the ED was fair agreement. The reason for this situation may be the uncommon presence of pneumomediastinum and insufficient experience in tomographic evaluation. Less than 50% of the patients with pericardial effusion could also be diagnosed; this may be because of emergency physicians' use for evaluating pericardial effusion with echocardiography or USG with e-FAST protocol and do not pay attention to CT imaging. Attention should be paid to evaluate thoracic pathologies, such as pericardial effusion, pneumomediastinum, and lung contusion in the ED.

It is noticeable that accurate diagnosis of solid organ injuries to splenic injuries in abdominal CT imaging results is higher and more consistent than others. The spleen is a solid organ frequently injured in blunt abdominal trauma [11]. In this perspective, it is important to be diagnosed correctly. It was found that the rates of correct diagnosis and agreement levels with RD in patients with mesenteric and bladder injury, which are among the abdominal organ pathologies, were lower than other diagnoses. We think that it would be beneficial for evaluators to be more careful in these matters and to report the imaging by radiology quickly. Abdominal CT is more difficult to assess than CTs of other regions by clinicians. Due to the presence of more organs and the complexity of the images, more overlooked diagnoses can be expected. Indeed, in Yang's study, diagnoses on abdominal imaging were missed more (53%). Thirty percent of the diagnoses missed in our study were abdominal CT-related pathologies. It shows that this rate is lower than that in the literature, which suggests that attention is paid to intra-abdominal pathologies.

In 76 patients with a pelvic fracture, the rates of diagnosing any pelvic fracture and overlapping with RD were higher than examining pelvic fractures one by one. Hence, we think that pelvic fracture is recognized, but it was named wrong. In our study, in evaluating the EDD process, a general EDD was evaluated without separating the opinion of the emergency physician and consultant physician. In this sense, we think that both emergency and orthopedic physicians should be supported with education in order to name pelvic fractures correctly.

In our study, additional medical intervention was performed in 13 patients (5.3%), and additional surgical intervention was performed in eight patients (3.3%) due to the diagnoses missed in the ED. In our opinion, it is very important to make correct diagnoses in the emergency room processes, and due to the chaotic processes of emergency services, patients should be evaluated in detail in the clinic where they are hospitalized. In Vles et al.'s study, it was mentioned that 27 (55%) of 49 patients with a missed diagnosis required an additional intervention and 12 patients (25%) needed surgical interventions [7]. In the study of Houshian et al., 27 (31%) of 86 missed diagnoses required additional surgical intervention. The rate of missed diagnosis in our patients was consistent with the literature. The proportion of missed diagnoses affecting the treatment processes in patients and the need for additional medical intervention were lower than those in the literature. This result shows that we pay more attention to major and fatal diagnoses, and we skip more minor diagnoses that will not affect treatment processes. Focusing on major diagnoses may have caused minor diagnoses to be overlooked or neglecting in recording minor diagnoses in the patient file because it did not affect the treatment process.

In this study, we could not determine the effect of missing radiological diagnosis as an indicator on mortality and morbidity in patients. However, it may still be useful to perform tertiary examination to re-evaluate missed diagnoses [12]. There are many articles in the literature that emphasize the mortality- and morbidity-reducing aspect of tertiary care [13-16].

Limitations

Retrospective file scanning was performed in our study. Although some pathologies are observed, they may not be recorded in the electronic file system. We think that the records can be incomplete, especially in patients with pathologies that are not life-threatening or that can be evaluated as minor because of focusing more important pathologies.

## Conclusions

According to the results of our study, there was a substantial agreement between EDDs and RDs in patients with multiple trauma who got full-body CT. The rate of missed diagnosis in full-body CT in our ED is 18.3%. Thoracic region pathologies are the most frequently missed pathologies, and orthopedics was the most frequently consulted department related to the missed diagnoses. Patients who have a missed diagnosis had lesser discharging from the ED than the other patients and had higher rates of in-hospital deaths, hospitalization, and intensive care unit admission.

## References

[1] Janjua KJ, Sugrue M, Deane SA (1998). Prospective evaluation of early missed injuries and the role of tertiary trauma survey. J Trauma.

[2] Yang F, Bai X, Li Z (2011). Analysis of misdiagnosis in patients with multiple trauma. Chin J Traumatol.

[3] Buduhan G, McRitchie DI (2000). Missed injuries in patients with multiple trauma. J Trauma.

[4] Causey MW, Oguntoye MO, Miller S, Andersen C, Singh N (2010). Limb salvage after delayed diagnosis for blunt traumatic infrapopliteal occlusion. J Vasc Surg.

[5] Vadivelu S, Bell RS, Crandall B, DeGraba T, Armonda RA (2010). Delayed detection of carotid-cavernous fistulas associated with wartime blast-induced craniofacial trauma. Neurosurg Focus.

[6] Pfeifer R, Pape HC (2008). Missed injuries in trauma patients: a literature review. Patient Saf Surg.

[7] Vles W, Veen E, Roukema J, Meeuwis J, Leenen L (2003). Consequences of delayed diagnoses in trauma patients: a prospective study. J Am Coll Surg.

[8] Kalemoglu M, Demirbas S, Akin ML, Yildirim I, Kurt Y, Uluutku H, Yildiz M (2006). Missed injuries in military patients with major trauma: original study. Mil Med.

[9] Banaste N, Caurier B, Bratan F, Bergerot JF, Thomson V, Millet I (2018). Whole-body CT in patients with multiple traumas: factors leading to missed injury. Radiology.

[10] Houshian S, Larsen MS, Holm C (2002). Missed injuries in a level I trauma center. J Trauma.

[11] Martin JG, Shah J, Robinson C, Dariushnia S (2017). Evaluation and management of blunt solid organ trauma. Tech Vasc Interv Radiol.

[12] Enderson B, Reath D, Meadors J, Dallas W, DeBoo J, Maull K (1990). The tertiary trauma survey: a prospective study of missed injury. J Trauma.

[13] Soundappan SV, Holland AJ, Cass DT (2004). Role of an extended tertiary survey in detecting missed injuries in children. J Trauma.

[14] Hoff WS, Sicoutris CP, Lee SY (2004). Formalized radiology rounds: the final component of the tertiary survey. J Trauma.

[15] Huynh TT, Blackburn AH, McMiddleton-Nyatui D, Moran KR, Thomason MH, Jacobs DG (2010). An initiative by midlevel providers to conduct tertiary surveys at a level I trauma center. J Trauma.

[16] Okello CR, Ezati IA, Gakwaya AM (2007). Missed injuries: a Ugandan experience. Injury.

